# The Airway Pathobiome in Complex Respiratory Diseases: A Perspective in Domestic Animals

**DOI:** 10.3389/fcimb.2021.583600

**Published:** 2021-05-14

**Authors:** Núria Mach, Eric Baranowski, Laurent Xavier Nouvel, Christine Citti

**Affiliations:** ^1^ Université Paris-Saclay, Institut National de Recherche Pour l’Agriculture, l’Alimentation et l’Environnement (INRAE), AgroParisTech, Génétique Animale et Biologie Intégrative, Jouy-en-Josas, France; ^2^ Interactions Hôtes-Agents Pathogènes (IHAP), Université de Toulouse, INRAE, ENVT, Toulouse, France

**Keywords:** domestic animals, pathobiome, respiratory infectious diseases, airway microbiota, public health

## Abstract

Respiratory infections in domestic animals are a major issue for veterinary and livestock industry. Pathogens in the respiratory tract share their habitat with a myriad of commensal microorganisms. Increasing evidence points towards a respiratory pathobiome concept, integrating the dysbiotic bacterial communities, the host and the environment in a new understanding of respiratory disease etiology. During the infection, the airway microbiota likely regulates and is regulated by pathogens through diverse mechanisms, thereby acting either as a gatekeeper that provides resistance to pathogen colonization or enhancing their prevalence and bacterial co-infectivity, which often results in disease exacerbation. Insight into the complex interplay taking place in the respiratory tract between the pathogens, microbiota, the host and its environment during infection in domestic animals is a research field in its infancy in which most studies are focused on infections from enteric pathogens and gut microbiota. However, its understanding may improve pathogen control and reduce the severity of microbial-related diseases, including those with zoonotic potential.

## Introduction

Complex respiratory diseases are highly prevalent and can be life threatening in domestic animals in which a prompt diagnosis and targeted treatments are essential (Ericsson et al., 2016; Bond et al., 2017; Oladunni et al., 2019; Ericsson et al., 2020). Besides impacting animal health and welfare, they cause a significant health burden worldwide including high treatment costs, high morbidity, premature mortality, decreased performance and severe consequences to public health and the environment (Kuiken et al., 2005; Holt et al., 2011; Ericsson et al., 2016; Bond et al., 2017; Oladunni et al., 2019; Blakebrough-Hall et al., 2020; Ericsson et al., 2020). For example, the bovine respiratory disease complex (BRDC) is a leading cause of morbidity and economic losses in wealthy countries which ranges from 30% in Belgium (van Leenen et al., 2020) to 49% in Switzerland and up to 80% in the U.S.A. (Hilton, 2014). Similarly, the porcine respiratory disease complex (PRDC) in finishing pigs continues to grow (Qin et al., 2018) with a morbidity rate ranging from 10% in Denmark (Hansen et al., 2010) to 40% in the U.S.A (Harms et al., 2002). As for common livestock animals, the equine respiratory disease complex (ERDC) is an important respiratory infection in horses (Wasko et al., 2011) that affects up to 66% of the equine population (Wasko et al., 2011) and can quickly disqualify a horse from racing, showing, training or other activities for periods of time ranging from a few days to months. In companion animals, the canine infectious respiratory disease complex (CIRDC) affects 66% of the dogs studied in Europe, including both peat and kenneled dogs (Mitchell et al., 2017). The feline respiratory disease complex has been described as one of the most importance cause of morbidity for cats in U.S.A, with reported incidence as high as 30% (Wagner et al., 2018). Finally, the respiratory disease complex in poultry remains widespread and has become endemic in different countries causing subclinical infections, mild respiratory symptoms and high production losses in birds either raised for meat or eggs (Awad et al., 2014; Guabiraba and Schouler, 2015; Patel et al., 2018; Samy and Naguib, 2018).

Our view on the dynamics of airway diseases has now been broadened to include an additional aspect of the complex system: the microbiota of the host and its environment (Bernardo-Cravo et al., 2020). The properties of the host microbiome have led to the concept of the holobiome in which the host and its microbial communities are merged into a symbiotic superorganism and later, to the concept of pathobiome to further consider microbiome communities in disease state (Vayssier-Taussat et al., 2014). The importance of the pathobiome concept arose from human studies in which disruption of a health-promoting and stable gut microbiome results in dysbiosis– a microbiome community acting as pathogenic entity (Bass et al., 2019). In this concept, the pathogen and host microbiome are assembled, leading to similar issues raised for the holobiome (Bernardo-Cravo et al., 2020).

Much work is required to identify the mechanisms underlying the microorganism relations and perturbations of a balanced and healthy microbiome that lead to a pathobiome (Bass et al., 2019). Commensal microbiome might act as a gatekeeper that provides resistance to infection on the mucosal surface and spreading to the lungs (Man et al., 2017; Li et al., 2019) or enhance disease exacerbation.

The number of studies addressing the role of the respiratory bacterial communities in animal health and disease is limited and almost entirely restricted to animals farmed for foods such as cattle and pigs. Moreover, although microbiota is composed of bacteria, protists, fungi, archaea and viruses, most studies in domestic animals have focused solely on bacterial microbiota.

In cows and feedlot cattle, several studies have reported how a healthy respiratory microbiota is established in the airways and surveyed which host and environmental factors drive it (Timsit et al., 2016; Hall et al., 2017; Holman et al., 2017; Nicola et al., 2017; Zeineldin et al., 2017a; Holman et al., 2018; Stroebel et al., 2018; Amat et al., 2019; McMullen et al., 2020; Zeineldin et al., 2020a). Analogously, predicting when and how respiratory microbiome breaks down is at the heart of several studies in swine (Cortes et al., 2018; Zeineldin et al., 2018; Jakobsen et al., 2019; Megahed et al., 2019; Mou et al., 2019; Wang et al., 2019; Pirolo et al., 2021). For instance, it appears as though that during weaning, several microorganisms act synergistically to mediate the BRDC (Gaeta et al., 2017; Klima et al., 2019; McMullen et al., 2019; Zeineldin et al., 2019) and PRDC (Wang et al., 2018; Li et al., 2021). Aside from ruminants and swine, efforts have focused on characterizing the composition patterns of the upper and lower airways in sheep (Glendinning et al., 2016; Glendinning et al., 2017a), horses (Bond et al., 2017), animals domesticated for companionship (Ericsson et al., 2016; Vientoos-Plotts et al., 2017; Vientós-Plotts et al., 2017; Fastrès et al., 2019; Fastrès et al., 2020b) and commercial birds (Shabbir et al., 2014; Glendinning et al., 2017b; Johnson et al., 2018; Ngunjiri et al., 2019; Abundo et al., 2020; Taylor et al., 2020; Abundo et al., 2021; Kursa et al., 2021).

Determining the underlying causes of respiratory illness is complicated. Species of veterinary interest are subjected to different host variables, environments and pathogens, which could all play a role in disease, either alone or in concert. However, we show evidence supporting the existence of an interplay between respiratory pathogens, commensal microbiota, host and environment in the respiratory apparatus of domestic animals. We untangle whether the airway microbiota act as a gatekeeper that provides resistance to pathogen colonization, thus ensuring resiliency and health in domestic animals. Moreover, we provide an overview of the covariation between airway microbiota, host and environment factors within and between species and we suggest potential applications of this knowledge in veterinary medicine. Lastly, we outline avenues to understand if gut microbiota-derived metabolites could be key molecular mediators of the microbiota-gut-lung axis and affect the onset of the respiratory diseases. Thus, the exploration of the relationships between respiratory pathogens, hosts and respiratory microbiota in domestic animals is timely and novel.

## Do Differences in the Respiratory System Contribute to Microbiome Composition? Mammals *Versus* Birds

**Figure 1:**
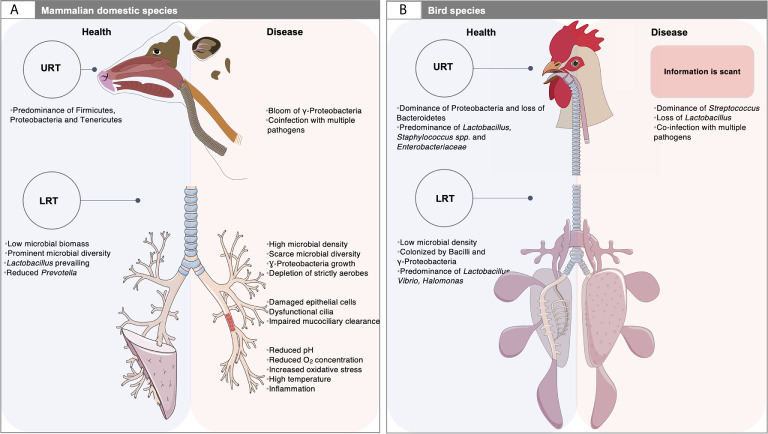
Airway microbiota: a matter of animal species, physiology and diseased settings. **(A)** The respiratory tract of domestic mammals is mainly colonized by Firmicutes, Proteobacteria, Tenericutes and Bacteroidetes. At steady state (left, blue rectangle), the microbial biomass in the low respiratory tract (LRT) is low and likely depends on the balance between migration of bacteria from the upper respiratory tract (URT) and the mucociliary and immune clearance. During respiratory disease (right; red rectangle), the LRT bacterial propagation outpaces the capacity of the airways to clear the microorganisms, which often results in increased microbial density and dysbiosis. Dysfunctional cilia and enhanced mucus production may contribute to reduced clearance and trapping of microorganisms within airways. An outgrowth of γ-Proteobacteria and loss of Firmicutes is observed in mammals, together with a limited microbial diversity, and the exacerbation of respiratory inflammation and loss of mucosal barrier function. In this context, co-infections by other pathogens or pathobionts are frequent. This polymicrobial disease involves microorganisms that act synergistically, or in succession to mediate complex disease processes. Studying such questions is challenging due to the complexity of sample collection. The exploration of LRT microbiota is based on invasive sampling techniques such as bronchoalveolar lavage, bronchus mucosal scraping and tracheal aspirates. Swaps of the deep nasal cavity, nasopharynx, oropharynx, paranasal sinus and tonsil are usually taken to explore the URT site; **(B)** The URT of birds is qualitatively similar to that of mammals. However, the nasal and oral cavities communicate with each other through the choanal cleft region. Their URT is mainly colonized by Firmicutes and Proteobacteria. At the genus level, a healthy ecosystem mainly includes highly dominant taxa, namely *Lactobacillus, Staphylococcus* spp. and members of the *Enterobacteriaceae* family. Unlike mammals, the LRT comprises the syrinx and the air sacs distributed throughout the body. In a stable state, the microbial density in the lung is very low. The LRT is mainly colonized by Bacilli and γ-Proteobacteria. At the genus level, *Lactobacillus* is the dominant bacterial taxon, followed by *Vibrio* and *Halomonas*. Yet, information related to the microbiota composition under respiratory diseases is scant. The assessment of microbiota in the URT is based on choanal swabs, nasal cavity wash and upper and lower tracheal washes, whereas the description of the LRT microbiota depends on lower respiratory lavage. The picture of mammalian LRT was downloaded from smart Servier Medical Art https://smart.servier.com without changes. Servier Medical Art by Servier is licensed under a Creative Commons Attribution 3.0 License. Written permission for publication of the chicken figure and chicken respiratory system drawing has been taken.

It is remarkable that mammals and birds, the two great classes of vertebrates capable of sustained high oxygen consumption, present many distinct differences (morphologic, physiologic and mechanic) in the respiratory tract (West et al., 2007) (**Figure 1**). For example, in mammals, the lower respiratory tract (LRT) comprises the trachea, the primary bronchi and the lungs, whereas in birds it involves the syrinx, the air sacs distributed throughout the body, the bronchi, the bronchioles and lungs (Nochi et al., 2018; Abundo et al., 2020; Abundo et al., 2021). Unlike mammals, the avian lungs are essentially rigid and tubular without alveoli (Bernhard et al., 2004) and have a unidirectional airflow in which the lungs are ventilated *via* air sacs (Nochi et al., 2018). By contrast, the mammalian lungs have reciprocating ventilation with large terminal air spaces (alveoli) and reduced airflow in peripheral structures (Bernhard et al., 2004). Additionally, the blood-gas barrier of the avian lung is approximately 56-67% thinner than that of a mammal of the same body mass while the respiratory surface area is approximately 15% greater (Maina et al., 1989), which altogether have the potential for more efficient gas exchange (West et al., 2007). As a result, the environment of the LRT is very different between mammal and birds (West et al., 2007). The upper respiratory tract (URT) of birds is qualitatively similar to that of mammals. It has a nasal cavity with communicating sinuses, a larynx supported by cartilaginous plates and the trachea, although the nasal and oral cavities communicate with each other through the choanal cleft region, which appears in birds to be related to the incomplete secondary palate (**Figure 1**).

These host factors that themselves change across birds and mammals likely play an important role in structuring the airway microbiomes across species. It is possible that some other aspects of adaptation to flight have a net effect on microbiome composition, diversity and function in birds. For instance, the large mass-specific gas uptake by the avian respiratory system (Brown et al., 1997), as well as the large trachea length, the decreased body mass, the higher metabolic rate, the higher average body temperature (40-41°C) and the higher resting heart rate (~245 beats/min) (Brown et al., 1997) probably influence the airway microbiome. The reduction in genome size compared to all amniotes, coupled with a shrinkage of large numbers of genes involved in the immune function (Lovell et al., 2014) might also consistently impact the microbiome composition and function.

## Host Determinants in a Microbe-Dominated World: A Matter of Species

The importance of host-related factors to the diversity of respiratory microbiomes is still poorly understood. However, we have observed that more closely related mammals tend to present a similar degree of taxa composition in their microbiomes, with likely downstream effects on host immunity and metabolic potential (**Table 1**). This may be due to the breathing patterns in mammals and the fact that the anatomy and physiology of both the upper and lower respiratory tract do not differ greatly among mammal species (**Figure 1**). In healthy bovine individuals, the URT microbiota (based on nasal and nasopharyngeal swab samples) is dominated by Proteobacteria, a group of bacteria suggested to have high functional variability (Bradley and Pollard, 2017), followed by Tenericutes, Firmicutes and Bacteroidetes (Gaeta et al., 2017; McMullen et al., 2019). Proteobacteria, Firmicutes, and Bacteroidetes are the three most dominant phyla in the URT bacterial communities in piglets (Slifierz et al., 2015; Correa-Fiz et al., 2016; Wang et al., 2019). Precisely, the composition of the tonsillar and oropharyngeal microbiome of adult pigs resembled that of the nasal microbiota, with Proteobacteria and Firmicutes as the predominant phyla (Lowe et al., 2012; Wang et al., 2018). Similarly, the URT microbiome of healthy horses is mainly dominated by Proteobacteria, Actinobacteria, Firmicutes, and Bacteroidetes (Bond et al., 2017). The most common phyla colonizing the nasal cavity of healthy dogs are Proteobacteria, followed by Firmicutes and Bacteroidetes (Tress et al., 2017). Proteobacteria is also the most abundant phylum in the cat respiratory tract, reaching up to 60% and 62% in the oropharyngeal swabs and BAL, respectively (Vientoos-Plotts et al., 2017).

**Table 1:** Recent metagenomic studies on the respiratory microbiota of domestic animals.

*Specie*		*Cohort description*	*Sample type*		*Platform and sequenced region*	*Main phyla*	*Genera*	*α-diversity*	*Reference*
*Bovine*	Healthy Holstein calves (n=32) *vs* calves with pneumonia (n=16), otitis (n=28) or both diseases (n=5). Time series; d3, d24, d28 and d35 of life. Age: 3 to 35 days of age			NPS	Illumina Miseq (V4)	Proteobacteria (30%-80%), Tenericutes (1%-10%), Firmicutes (1-20%), Bacteroidetes (1-10%)	*Mannheimia* (3-25%), *Mycoplasma* (3-18%), *Moraxella* (2-18%), *Psychrobacter* (3-14%), *Pseudomonas* (1-13%). Abundances of *Mannheimia, Moraxella*, and *Mycoplasma* were significantly higher in diseased *vs* healthy animals	α-diversity indices were not different between healthy and diseased calves	(Lima et al., 2016)
*Bovine*	Angus-beef cattle from weaning to 40 days after arrival at a feedlot (n=30). Time series: weaning, d0, d40			DNS	Illumina Miseq (V3)	Tenericutes (53.2%), Proteobacteria (34.7%), Firmicutes (4.2%), Bacteroidetes (3.7%), Actinobacteria (3.4%)	*Mycoplasma* (*M. dispar* and *M. bovirhinis*, 52%), *Pasteurella* (11%), *Ralstonia* (10%), *Moraxella* (9%)	No differences in α-diversity over time	(Timsit et al., 2016)
*Bovine*	Healthy Holstein heifer calves at 14d (n=10) and 28d (n=19) *vs* BRDC at 14d (n=6) and 28d (n=6). Age: 3 to 35 days of age			NPS	Illumina Miseq (Not specified)	Proteobacteria, Firmicutes, Actinobacteria, Bacteroidetes, Tenericutes	Healthy: *Mannheimia haemolytica* (7%), *Psychrobacter* (2-5%), *Actinobacillus* (3-6%), *Mycoplasma* (2-12%), BRDC: *Pseudomonas fluorescens* (0.02 – 13%), *Mycoplasma* (8%), *Mannheimia haemolytica* (6%), *Eubacterium* (6%), *Psychrobacter* (5%). No significant differences were detected between groups	Not specified	(Gaeta et al., 2017)
*Bovine*	Healthy post-weaned Piedmontese calves (n=11) *vs* calves with respiratory diseases (n=8)			DNS, TA	Illumina Miseq (V4)	DNS: Proteobacteria (36.1%), Tenericutes (27.7%), Firmicutes (18.4%), Bacteroidetes (10.1%), Actinobacteria (6.3%) TA: Tenericutes (78%), Proteobacteria (11%), Fusobacteria (4%), Bacteroidetes (4%), Actinobacteria (3%)	DNS: *Mycoplasma* (27%), *Moraxella* (17%), *Aggregatibacter* (4%), *Sphingomonas* (3%), *Corynebacterium* (1.3%), *Psychrobacter* (1.2%). TA: *Mycoplasma* (77%), *Pasteurella multocida* (7%), *Mannheimia* (1.6%), *Bacteroides* (1.5%), *Ureaplasma* (1.3%). No differences between healthy and the BRDC-affected calves	α-diversity indices were different between TA and DNS samples	(Nicola et al., 2017)
*Bovine*	Angus weaned beef calves fed with Selenium-biofortified alfalfa hay (Se) for 9 weeks (n=30) *vs* control (n=15). Age: 6.5 to 9 months old			DNS	Illumina Miseq (V4)	Control group: Proteobacteria (44%), Bacteroidetes (24%), Firmicutes (19%); Se group: Bacteroidetes (29%), Proteobacteria (27%), Firmicutes (23%)	Control group: *Moraxellaceae* (19%), *Chitinophagaceae* (13%), *Aggregatibacter* (11%), *Weeksellaceae* (7%) Se group: *Chitinophagaceae* (23%), *Moraxellaceae* (9%) *Ruminococcaceae* (6%)	Se group tended to have an enriched nasal microbiota	(Hall et al., 2017)
*Bovine*	Angus × Hereford heifers transport to a feedlot (n=14). Time series: d0, d2, d7 and d14. Age: 8 months old			NPS	Illumina Miseq (V4)	Firmicutes (22-42%), Proteobacteria (10-30%), Tenericutes (5-20%), Actinobacteria (5-12%)	*Mycoplasma* (0.003-86%), *Psychrobacter* (0.55-90%), *Clostridium, Flavobacterium, Bacteroides, Rikenellaceae, Mannheimia, Acinetobacter, Corynebacterium, Moraxella, Pasteurella, Streptococcus*	Richness increased following feedlot placement (d2)	(Holman et al., 2017)
*Bovine*	Healthy Charolais calves (n = 8). Age: 6 to 8-month-old			DNS, BALF	Illumina Miseq (V3-V4)	DNS: Actinobacteria (43.9%), Proteobacteria (15.9%), Tenericutes (12.1%), Bacteroidetes (8.8%), Fusobacteria (0.1%) BALF: Proteobacteria (33.3%), Bacteroidetes (18.2%), Tenericutes (8.8%), Actinobacteria (4.9%), Fusobacteria (3.4%)	DNS: *Rathayibacter* (24.41%), *Mycoplasma* (12.20%), *Corynebacterium* (10.37%), *Clostridium* (2.53%), *Prevotella* (0.28%) BALF: *Mycoplasma* (9.48%), *Bibersteinia* (14.41%), *Prevotella* (11.69%), *Clostridium* (6.85%), *Rathayibacter* (0.07%)	No differences in α-diversity indices between DNS and BALF samples	(Zeineldin et al., 2017b)
*Bovine*	Healthy weaned feedlot cattle (n=60) *vs* cattle with bronchopneumonia (BP; n=60). Mixed-breed beef steers. Body weight: ~260 kg			NPS, TA	Illumina Miseq (V4)	Tenericutes (47%), Proteobacteria (26%), Firmicutes (21%)	TA healthy group: *Mycoplasma* (46%), *Lactococcus* (18%), *Histophilus* (10%), *Moraxella* (6%), *Mannheimia* (6%) Only in NPS healthy group: *Corynebacterium* (0.54%), *Jeotgalicoccus* (0.69%), *Psychrobacter* (0.52%), *Planomicrobium* (0.43%) NPS, TA of BP group: *Mycoplasma bovis* (46%), *Mannheimia haemolytica* (5.6%), *Pasteurella multocida* (2.18%)	Lower α-diversity in the NPS and TA of BP *vs* control	(Timsit et al., 2018)
*Bovine*	Weaned Angus-cross beef heifers transported directly to a feedlot (RANC; n=30) *vs* heifers co-mingled at an auction market for 24 h before being placed in a feedlot (AUCT; n=30). Time series: d0, d2, d7 and d30 after arrival			NPS, TA	Illumina Miseq (V4)	NPS: Tenericutes (41%), Proteobacteria (32%), Firmicutes (5%) TA: Tenericutes (50%), Proteobacteria (14%), Firmicutes (9%), Actinobacteria (4%)	NPS*: Mycoplasma* (40.8%), *Moraxella* (18.7%), *Pasteurella* (6.8%), *Mannheimia* (3.8%) TA: *Mycoplasma* (50.4%), *Pasteurella* (5.7%), *Moraxella* (3.1%)	No differences in α-diversity between RANC and AUCT, but it decreased over time	(Stroebel et al., 2018)
*Bovine*	Commercial feedlot cattle injected with antimicrobials (n=20) *vs* in-feed antibiotics (n=20). Age: 0 to 12 months			NPS	Illumina Miseq (V3-V4)	Not specified	*Psychrobacter* (20-55%), *Pseudomonas* (1-50%), *Mycoplasma* (1-15%), *Moraxella* (0-10%)	Non specified	(Holman et al., 2018)
*Bovine*	Healthy feedlot cattle (n=3) *vs* cattle with BRDC (n=15)			BALF	HiSeq 2500 (WMS)	Not specified	Healthy: *Bacillus* (6%), *Enterococcus faecium* (5%), *Brachyspira hampsonii* (4%), *Bacillus obstructivus* (3%), *Streptococcus pneumoniae* (2%) BRDC: *Mannheimia haemolytica* (18.8%), *Mycoplasma bovis* (5%), *Mycoplasma dispar* (3%), *Enterococcus faecalis* (2%), *Bacillus* VT-16-64 (2%)	Not specified	(Klima et al., 2019)
*Bovine*	Weaned Angus × Herford cross calves transported to feedlot (n=13). Time series: auction market (day 0), and feedlot placement (day 2, 7, 14)			NPS	Illumina Miseq (V4)	Proteobacteria (36.1%), Firmicutes (20.1%), Tenericutes (19.3%), Actinobacteria (12.7%), Bacteroidetes (8.6%)	*Acinetobacter* (1-4%), *Bacteroides* (1-2%), *Bifidobacterium* (0-2%), *Corynebacterium* (0-9%), *Jeotgalicoccus* (1-2%), *Mannheimia* (0-1%), *Methanobrevibacter* (1-5%), *Moraxella* (0-10%)	Increase of richness following transport to an auction market and feedlot	(Amat et al., 2019)
*Bovine*	Angus × Herford steers transported to a feedlot and injected with antimicrobials (n=12) *vs* in-feed antibiotics (n=12) and untreated (n=12). Time series: d0, d2, d5, d12, d19 and d34. Age: weaned calves			NPS	Illumina Miseq (V4)	Not specified	Untreated: *Acinetobacter* (2-5%), *Corynebacterium* (2-6%), *Jeotgalicoccus* (1-3%), *Moraxella* (1-20%), unclassified *Ruminococcaceae* (2-10%), *Mycoplasma* (1-20%), *Planomicrobium* (2.5-5%), *Psychrobacter* (0-10%)	Richness and α-diversity increased following transport to the feedlot	(Holman et al., 2019)
*Bovine*	Healthy calves (n=82) *vs* BRDC calves (n=82). Time series: 3-12d, 13-20d, 21-44d. Animals were raised without antimicrobials. Age: newly received feedlot cattle of about ~218 kg			DNS	Illumina Miseq (V4)	Proteobacteria (69%), Tenericutes (23%), Firmicutes (3%), Actinobacteria (2%), Bacteroidetes (2%)	Healthy: *Moraxella* (22%), *Mycoplasma* (17%), *Histophilus* (16.62%) BRDC group: *Mycoplasma* (27%), *Histophilus* (21%), *Moraxella* (18%)	Species richness was lower in BRDC compared to healthy	(McMullen et al., 2019)
*Bovine*	Healthy pre-weaned calves fed 2 different milk-feeding programs (n=40). Age: 3.5 ± 1.15 days of age. BW: 39.3 ± 4.25 kg			DNS	Illumina Miseq (V1-V3)	Tenericutes (29.5%), Firmicutes (19.3%), Actinobacteria (19%), Proteobacteria (16%), Bacteroidetes (11.5%), Fusobacteria (2.5%)	*Pseudoclavibacter (*13.8%), *Mycoplasma* (29.5%). Differences between feeding regimes were observed for *Streptococcaceae* family and *Histophilus*	No differences in α-diversity between treatments	(Maynou et al., 2019)
*Bovine*	Healthy calves (n=9) *vs* BRDC affected calves treated with tilmicosin (n=9). Age: 6 to 8 months of age.			NPS	Illumina Miseq (V1-V3)	Firmicutes (27.07%), Actinobacteria (24.51%)*, Tenericutes* (16.05%), and Proteobacteria (14.43%). BRDC-affected and treated calves showed significant decrease in Actinobacteria	*Mycoplasma* (18.73%)*, Microbacteriaceae* (9.36%)*, Acinetobacter* (7.35%), *Corynebacterium* (6.36%)	No differences in α-diversity between groups	(Zeineldin et al., 2020b)
*Sheep*	Suffolk cross sheep sampled at 3 spatially disparate segmental bronchi (n=6). Time series: day 0, 1 month, and 3 months. Age: 20 months old			SBT	Illumina Miseq (V2-V3)	Not specified	*Staphylococcus* (16%), *Corynebacterium* (9%), *Jeotgalicoccus* (5%), *Streptococcus* (5%)	No difference in richness or α-diversity between different lung sites	(Glendinning et al., 2016)
*Sheep*	Scottish mule x Suffolk sheep (n=40). Age: 7 weeks old			OPS, BALF	Illumina Miseq (V2-V3)	Not specified	OPS*: Pasteurellaceae (22%), Mannheimia (14%), Fusobacterium (11%), Bibersteinia trehalosi (8%), Neisseriaceae (7%), Moraxella (6%), Bibersteinia (5%)* BALF*: Staphylococcus equorum (13%), Staphylococcus sciuri (6%), Mannheimia (5%), Prevotella (5%)*	No difference in richness or α-diversity	(Glendinning et al., 2017a)
*Pig*	Healthy commercial pigs (n=30) *vs* pigs with Glässer’s disease (n=40). Age: 3-4 weeks old (before weaning)			DNS	Illumina Miseq (V3-V4)	Healthy: Proteobacteria (32.5%), Firmicutes (21.1%), Tenericutes (2.2%), Actinobacteria (1.3%) Glässer: Proteobacteria (44%), Firmicutes (18.5%), Tenericutes (2.2%), Actinobacteria (1.3%)	unclassified *Pasteurellaceae* (27.0%), *Moraxella* (17.2%), *Weeksella* (12.9%), *Haemophilus* (6.1%), Healthy: *Moraxella* (13.6%), *Enhydrobacter* (3.1%), *Haemophilus* (2.8%) Glässer: *Moraxella* (22.2%), *Haemophilus* (9.4%), *Enhydrobacter* (4.3%)	Healthy status was associated to higher species richness and α-diversity	(Correa-Fiz et al., 2016)
*Pig*	Piglets treated by Ceftiofur Crystalline free acid (CCFA, n=4), Ceftiofur hydrochloride (CHC, n=4), Tulathromycin (TUL, n=4), Oxytetracycline (OTC, n=4), or Procaine Penicillin G (PPG, n=4). Time series: day 0, 1, 3, 7, and 14 after dosing. Age: 8-week-old			DNS	Illumina Miseq (V1-V3)	Firmicutes (46.46%), Proteobacteria (31.87%), Bacteroidetes (9.64%)	*Moraxella* (21.52%), *Clostridium* (19.74%), *Streptococcus* (10.93%), *Calothrix* (5.43%), *Prevotella* (4.49%)	The α-diversity was not affected by treatment	(Zeineldin et al., 2018)
*Pig*	Cross-bred Yorkshire x Hampshire healthy newborn piglets (n=28). Time series: 8h post-birth, 1, 2, 3 and 4 weeks of age			TS	Illumina Miseq (V4)	Not specified	Post-born piglets were colonized by *Streptococcus, Staphylococcus, Moraxella, Rothia*, and *Pasteurellaceae*. By 1 week of age, members of the *Pasteurellaceae, Moraxellaceae*, and *Streptococcaceae* families were the most dominant	Not specified	(Cortes et al., 2018)
*Pig*	Healthy cross-bred pigs (n=10) *vs* pigs with Porcine respiratory disease (PRDC) disease (n=23). Age: 2-3 weeks of age (before weaning)			NPS	Illumina Miseq (V3-V4)	Firmicutes (53.11%), Proteobacteria (27.89%), Bacteroidetes (12.17%), Fusobacteria (3.15%), Actinobacteria (2.29%)	Healthy: *Lactobacillus* (30.44-37.28%), *Streptococcus* (23.60-32.26%), *Actinobacillus* (11.48-15.09%), *Bergeyella* (3.10%), *Escherichia-Shigella* (2.24%) PRDC: *Streptococcus* (22.22-26.25%), *Actinobacillus* (9.66-17.63%), *Moraxella* (12.02-15.82%), *Veillonella* (7.16%), *Bergeyella* (4.60-7.46%), *Fusobacterium* (4.64%), *Porphyromonas* (4.31%), *Escherichia-Shigella* (4.19%)	No differences in α-diversity and richness between the healthy and PRDC groups	(Wang et al., 2018)
*Pig*	Healthy Duroc Landrace Yorkshire cross-breed piglets (n=10) *vs* piglets challenged with Porcine reproductive and respiratory syndrome (PRRSV) (n=10). Age: 8 to 10 weeks of age			BALF	Illumina Miseq (V3-V4)	Healthy: Firmicutes (79.8–89.8%), Tenericutes (0.18-2.4%), Proteobacteria (5.3-13.4%) PRRSV: Proteobacteria (40.8-49%), Firmicutes (2.4–8.8%), Tenericutes (27-40.0%)	Healthy: *Anoxybacillus, Caloramator* PRRSV: *Haemophilus parasuis* (35–48%), *Mycoplasma hyorhinis* (27–41%), *Bacteroides* (4–11%), *Chloroplast* (1–3%), unclassified *Chitinophagaceae* (1–7%)	Reduced α-diversity in the PRRSV group	(Jiang et al., 2019)
*Pig*	Cross-bred pigs with lung-lesion and raised under natural conditions (n=20). Age: 240 days of age			BALF	Illumina Miseq (V3-V4)	Proteobacteria (34.2%), Tenericutes (22.3%), Bacteroidetes (18.8%), Firmicutes (18.1%)	*Mycoplasma* (13.0%), *Methylotenera* (10.9%), *Ureaplasma* (9.2*%), Phyllobacterium* (5.3%), *Prevotella* (4.0%), *Sphingobium* (3.2%), *Lactobacillus* (3.0%), *Thermus* (2.7%), *Streptococcus* (2.4%), *Haemophilus* (1.8%)	Reduced α-diversity in lungs with higher level of lesions	(Huang et al., 2019)
*Pig*	Commercial healthy pigs at slaughter (n=10)			TS	Illumina Miseq (V3-V4)	Proteobacteria (30–40%), Firmicutes (30%), Fusobacteria (20%), Bacteroidetes (10–20%). No differences between tonsil surface and in deep tonsil tissue	Surface of tonsils: *Actinobacillus* Deep tonsils: *Yersinia, Pasteurella*.	No difference in α-diversity between the surface and the deep tonsil tissue	(Jakobsen et al., 2019)
*Pig*	Duroc×Landrace×Yorkshire growing pigs exposed to different levels of gaseous ammonia (n=72). Body weight ∼30 kg			DNS, TS	Illumina Miseq (V3-V4)	Proteobacteria (36.4%), Firmicutes (34.8%), Bacteroidetes (19.9%), Actinobacteria (4.1%)	*Moraxella, Pseudomonas, Lactobacillus, Prevotella, Bacteroides, Megasphaera, Streptococcus, Rothia, Allobaculum, Blautia, Oscillospira*	Ammonia concentration decreased the α-diversity	(Wang et al., 2019)
*Pig*	Commercial weaned pigs housed to simple slatted-floor (S, n=75) *vs* complex straw-based rearing ecosystem (C, n=75). Sampling time: from 164 days post-weaning at the time of slaughter			BMS	Illumina Miseq (V3-V4)	Firmicutes (58.2%), Proteobacteria (30%) Actinobacteria phylum was more abundant in C-raised pigs compared to S-raised pigs	*Bacteroidetes, Clostridium*, *Streptococcus* *Anaerotruncus* (8%) was higher in S-raised pigs whereas *Bacteroidetes* (24.3%) and *Chitinophagaceae* (15.4%) were higher in C-raised pigs	The S ecosystem increased the α-diversity	(Megahed et al., 2019)
*Pig*	Post-weaned pigs with oxytetracycline parenterally administered (n=21) or in feed (n=22), and the non-medicated feed group (n=22). Time series: days 0 (before start of treatment), 4, 7, 11, and 14			DNS, TS	Illumina Miseq (V4)		DNS untreated: *Streptococcus* (23.61%), *Moraxellaceae* (29.74%), *Actinobacillus* (9.30%), *Moraxella* (3.36%), *Lactobacillus* (2.43%), DNS antibiotic: *Moraxellaceae* (32.84%), *Streptococcus* (12.68%), *Moraxella* (5.01%), *Lactobacillus* (2.15%) Tonsils untreated: *Veillonella* (18.27%), *Streptococcus* (16.31%), *Actinobacillus* (15.46%), *Bacteroides* (13.91%), *Fusobacterium* (6.95%) Tonsils antibiotic: Actinobacillus (22.90%), Bacteroides (17%), *Veillonella* (14.03%), *Streptococcus* (13.60%), *Fusobacterium* (7.09%)	Nasal α-diversity with antibiotic treatment was lower compared to control group. In tonsils, α-diversity was not affected by treatment	(Mou et al., 2019)
*Pig*	Healthy cross-bred pigs (n=30) *vs* pigs with Glässer’s disease, n=51). Age: weaning pigs (3-4 weeks of age)			DNS	Illumina Miseq (V3-V4)	Not specified	Glässer: *Corynebacterium, Clostridium* XI, *Escherichia/Shigella*, Healthy: *Odoribacter, Planobacterium, Phascolarctobacterium.*	Not specified	(Mahmmod et al., 2020)
*Pig*	Healthy duroc×Landrace×Yorkshire growing pigs (n=8) *vs* diseased pigs with PRDC (n=20). Age: 270 ± 3 days of age			BALF	Illumina Miseq (V3-V4)	Healthy: Proteobacteria (59%), Firmicutes (28.55%), Tenericutes (9.94%), Bacteroidetes (2%) Diseased: Proteobacteria (54%), Firmicutes (34%), Tenericutes (3%), Bacteroidetes (7%)	*Healthy: *Higher abundance of Lactococcus, *Enterococcus*, *Staphylococcus*, *Lactobacillus. *Diseased: Enhanced richness of *Streptococcus*, *Haemophilus*, *Pasteurella*, *Bordetella*	α-diversity was lower in healthy individuals than in diseased group	(Li et al., 2021)
*Bird*	Birds raised at 3 different farms in Pakistan (n=14)			TA, BALF	454 Roche	Proteobacteria, Firmicutes, Tenericutes, Actinobacteria, Bacteroidetes	Farm A: unclassified Ɣ Proteobacteria, Farm B: *Avibacterium* Farm C: unclassified *Enterobacteriaceae, Pseudomonas*	The α-diversity was higher in farms B and C than farm A	(Shabbir et al., 2014)
*Bird*	Healthy commercial chickens aged 2 days (n = 5), 3 weeks (n = 5) and 30 months (n = 6)			DNS, BALF	Illumina Miseq (V2-V3)		DNS: *Staphylococcus* (8.0%), *Lactobacillus* (6.2%), unclassified *Enterobacteriaceae* (6.0%), *Faecalibacterium prausnitzii* (5.0%), *Staphylococcus equorum* (5.0%), *Lactobacillus reuteri* (4.4%) BALF: *Pseudomonas* (20.7%), *Achromobacter* (4.8%), *Lactobacillus* (4.8%), *Turicibacter* (4.7%), SMB53 (3.6%). The 30-month-old birds showed lower *lactobacilli* but higher *Jeotgalicoccus*, *Staphylococcus* and smb53	The richness and α-diversity were different between DNS and BALF. Richness raised with age	(Glendinning et al., 2017b)
*Bird*	Commercial Coob 500 broilers from different flocks (n=120). Three grow-out cycles from 0 to 42 d of age. A cross-sectional sampling with different ages (n=90)			TA	Illumina Miseq (V4)	Not specified	*Lactobacillus, Escherichia/Shigella, Staphylococcus, Corynebacterium*, unclassified *Moraxellaceae*, unclassified *Ruminococcaceae*, unclassified *Clostridiales, Ruminococcus, Psychrobacter, Blautia*	Not specified	(Johnson et al., 2018)
*Bird*	Hy-Line W-36 commercial layers (n=181). Nine grow-out cycles from 5 weeks to > 17 weeks of age			DNS, TA	Illumina Miseq (V3-V4)	Not specified	DNS: *Staphylococcus, Enterobacteriaceae*, unclassified *Lactobacillaceae, Lactobacillus reuteri, Faecalibacterium prausnitzii, Deinococcus*, unclassified *Burkholderiaceae* TA: *Lactobacillus*, *Clostridium, Enterococcus, Escherichia-Shigella, Callibacterium, Irnithobacterium, Staphylococcus, Streptococcus.*	Not specified	(Ngunjiri et al., 2019)
*Bird*	Healthy commercial turkeys sampled at 3 time points during brooding (1, 3, and 5 weeks) and grow-out (8, 12, and 16 weeks; bn= 104).			DNS, TA	Illumina Miseq (V4)	DNS: Firmicutes and Actinobacteria TA: Proteobacteria	*Deinococcus*, *Corynebacterium*, and *Staphylococcus*	The α-diversity in the nasal cavity or trachea did not change with age	(Taylor et al., 2020)
*Bird*	Healthy ale Arbor Acres broilers exposed to 4 different levels of ammonia for 21 days (n=228). Age: 22 days of age			TA	Illumina Miseq (V3-V4)	Firmicutes (70%), Proteobacteria (15%)	*Lactobacillus* decreased under different levels of ammonia exposure, whereas *Faecalibacterium, Ruminococcus*, *unclassified Lachnospiraceae, Ruminococcaceae UCG-014, Streptococcus* and *Blautia* increased with ammonia levels	The α-diversity decreased with the ammonia levels	(Zhou et al., 2021)
*Horse*	Healthy horses (n=3) *vs* healthy horses treated with dexamethasone (n = 3) and *vs* horses with Inflammatory Airway Disease (IAD; n = 7).			DNS, BALF	Illumina Miseq (V3-V4)	Proteobacteria (43.85%), Actinobacteria (21.63%), Firmicutes (16.82%), Bacteroidetes (13.24%)	*Sphingomonas* (15.65%), *Pseudomonas* (14.57%), *Pantoea* (11.68%), *Knoellia* (2.80%), *Agrococcus* (2.61%) Arthrobacter (2.61%). *Streptococcus* was higher in IAD horses. Dexamethasone had no effect	No differences in α-diversity. Decrease in richness in BALF	(Bond et al., 2017)
*Horse*	Healthy adult horses (n=6) *vs* asthmatic horses (n=6). Animals were at pasture (low antigen exposure), housed indoors and fed good- quality hay (“moderate exposure”) or poor-quality hay (“high exposure”)			DNS, BALF	Illumina Miseq (Not specified)	DNS: Proteobacteria, Firmicutes BALF: Proteobacteria, Firmicutes, Bacteroidetes, Actinobacteria, Verrucomicrobia	DNS: *Pasteurella multocida* (39.5%), *Actinobacillus* (23.2%) BALF: *Enterobacteriaceae* (4%), *Pasteurella multocida* (3.0%), *Comamonadaceae* (2%), *Actinobacillus* (1.9%), *Staphylococcus* (1%)	DNS had higher richness and α-diversity than BALF samples	(Fillion-Bertrand et al., 2019)
*Horse*	Healthy horses (n=23) *vs* horses affected with primary (n=14) and secondary sinusitis (n=62). Age: 2 - 30 years old			PS	Illumina Miseq (V4)	Proteobacteria (63%, 23.7–99.0%), Firmicutes (14%, 0.38–64%), Actinobacteria (5.19, 0–62%)	Healthy*: Pseudomonas* (14.6%, 0.2–50.2%), *Delfta* (7.8%, 0–26.2%) *Stenotrophomonas* (6.7%, 0.1–18.2%) *Dokdonella* (5.1%, 0–80.8%) *Aggregatibacter* (4.7%, 0–71.0%) *Acinetobacter* (3.6%, 0–20.3%) *Achromobacter* (2.7%, 0–20.6%). Sinusitis: *Streptococcus equi* subsp. *Zooepidemicus*, *Fusobacterium*	No differences in Simpson diversity index between healthy and sinusitis groups	(Beste et al., 2019)
*Horse*	Healthy horses (n=4) *vs* horses with naturally occurring mild asthma (n = 16). Body weight ~435-612 kg Horses were treated with nebulized dexamethasone for 14 d.			NPS, BALF	Illumina Miseq (V4)	NPS: Proteobacteria (37.81%), Bacteroidetes (25.71%), Actinobacteria (17.77%), Firmicutes (17.9%)	*Hymenobacter* (16.51%), *Staphylococcus* (13.49%), *Pedobacter* (6.33%), *Moraxella* (4.50%), *Sphingomonas* (3.75%), *Pseudomonas* (3.62%)	Nebulized dexamethasone treatment decreased α-diversity in the nasopharynx	(Bond et al., 2020)
*Dog*	Healthy female dogs (n=16). Age: 2 - 8 years of age			DNS, NPS, BALF	Illumina Miseq (V4)	DNS: Proteobacteria (55.40%), Actinobacteria (0.73%) NPS: Proteobacteria (60.79%), Tenericutes (7.04%), Actinobacteria (0.41%) BALF: Proteobacteria (88.53%), Actinobacteria (2.61%)	DNS: unclassified *Pasteurellaceae* (7.20%), *Brevundimonas diminuta* (2.69%), Cardiobacteriales (2.11%) NPS: *Porphyromonas* (8.32%), Fusobacterium (2.96%), unclassified *Weeksellaceae* (2.70%), unclassified *Neisseriaceae* (3.36%) BALF: *Brevundimonas diminuta* (22.48%), *Sphingopyxis alaskensis* (2.37%), unclassified *Pasteurellaceae* (2.36%), *Propionibacteriaceae* unclassified (1.9%), unclassified *Bradyrhizobiaceae* (1.23%), unclassified *Methylobacteriaceae* (1.68%)	BALF in healthy dogs had equivalent richness to DNS	(Ericsson et al., 2016)
*Dog*	Healthy dogs (n = 23), dogs with malignant nasal neoplasia (n = 16), and dogs with chronic rhinitis (n = 8). Age ~6 years old			DNS	Illumina Miseq (V4)	Healthy: Proteobacteria (83.4%, 37.4%— 98.5%), Firmicutes (4.8%, 0.4–20.8%), Bacteroidetes (2.6%, 0.1–12.5%), Cyanobacteria (2.1%, 0.0–11.6%), Actinobacteria (2.1%, 0.1–8.6%).	Healthy group: *Moraxella* (59.2%), *Phyllobacterium* (3.4%), family *Cardiobacteriacea* (2.1%), *Staphylococcus* (1.7%). Diseased group: *Moraxella* (34.5%, 0.7–77.3%), order Streptophyta (6.4%, 0.0–16.6%), *Riemerella* (4.4%, 0.0–25.3%), family *Pasteurellaceae* (2.9%, 0.2–17.1%)	Shannon diversity index was lower for the healthy dogs than for the diseased dogs	(Tress et al., 2017)
*Dog*	Healthy client-owned dogs (n=5) *vs* client-owned dogs diagnosed with bacterial pneumonia (n=15)			OPS, BALF	Illumina Miseq (V4)	Not specified	OPS healthy: *Escherichia-Shigella* (15.33%), *Prevotella* (15.21%), *Streptococcus* (10.88%), *Bacteroides* (10.96%), *Mycoplasma canis* (7.67%), Acinetobacter (3.90%), BALF healthy: *Streptococcus* (96.4%)	Richness was decreased with pneumonia	(Vientós-Plotts et al., 2019)
*Dog*	Healthy research dogs (n=16) *vs* client-owned dogs diagnosed with Chronic Bronchitis (n=53). Age: between 8 and 9 years old			BALF	Illumina Miseq (V4)	Not specified	BALF healthy: *Pseudomonas stutzeri* (29.5%), *Acinetobacter* (20.9%), and *Brevundimonas* (20.1%) BALF diseased: *Acinetobacter* (22.3%), *Bradyrhizobium* (9.3%), *Brevundimonas* (8.3%), *Agrobacterium radiobacer* (7.1%)	Diseased dogs had lower richness than healthy research dogs	(Ericsson et al., 2020)
*Dog*	Healthy west highland white terriers (n=6) *vs* west highland white terriers with idiopathic pulmonary fibrosis (n=11)			BALF	Illumina Miseq (V1–V3)	Proteobacteria, Actinobacteria, Firmicutes, Bacteroidetes	Healthy group: *Cutibacterium, Staphylococcus, Streptococcus, Pseudomonas, Corynebacterium*, unclassified *Pasteurellaceae*, *Acinetobacter, Conchiformibius, Flavobacterium*, *Porphyromonas*. Diseased group: *Brochothrix, Curvibacter, Pseudarcicella*, unclassified *Flavobacteriaceae*	Lower α−diversity in diseased dogs compared to healthy	(Fastrès et al., 2020a)
*Dog*	Healthy dogs of different breeds (n=45) *vs* dogs diagnosed with idiopathic pulmonary fibrosis (n=11) exposed to different living conditions			BALF	Illumina Miseq (V1–V3)	Healthy: Proteobacteria, Actinobacteria, Firmicutes, Bacteroidetes	BALF healthy: *Cutibacterium, Staphylococcus, Streptococcus, Pseudomonas, Corynebacterium_1, Pasteurellaceae genus, Acinetobacter, Conchiformibius, Flavobacterium and Porphyromonas* (all together ~23% of bacterial population) BALF diseased: predominance of *Rhodoluna, Brochothrix, Curvibacter, Pseudarcicella*, *Flavobacteriaceae* family	No differences between living conditions for the α-diversity and the evenness. No differences between healthy and diseased dogs	(Fastrès et al., 2020b)
*Cat*	Healthy cats (n=6). Time series: day 0, week 2, and week 10. Age < 1 year old			OPS, BALF	Illumina Miseq (V4)	OPS Proteobacteria (60%, 54.78-74.28) BALF: Proteobacteria (62.36%, 27.26-81.65%)	OPS *Pasteurellaceae* (15.99%), *Moraxellaceae* (14.79%), *Porphyromonadaceae* (12.45%), *Pseudomonadaceae* (10.21%), *Paraprevotellaceae* (5.46%). BALF: *Pseudomonadaceae* (34.24%), *Sphingobacteriaceae* (22.56%), *Bradyrhizobiaceae* (15.86%).	OPS was richer than BALF. No differences in richness between time points	(Vientoos-Plotts et al., 2017)
*Cat*	Probiotic administration in healthy cats (n=6). Time series: baseline and after probiotic administration. Age < 1 year old			OPS, BALF	Illumina Miseq (V4)	Actinobacteria (59.27%), Firmicutes (40.67%)	*Bifidobacterium* (35.93%), *Bifidobacterium animalis* (23.29%), *Streptococcus* (25.53%), *Lactobacillus zeae* (11.64%), *Lactobacillus* (3.21%)	Probiotic increased the richness in the OPS and BALF	(Vientós-Plotts et al., 2017)
*Cat*	Healthy cats (n = 28) *vs* cats with nasal neoplasia (n = 16), and cats with feline upper respiratory tract disease (FURTD; n = 15). Age ranged from 6 months to 14.0 years			DNS	Illumina Miseq (V4)	Proteobacteria, Firmicutes, and Bacteroidetes	Healthy: *Moraxella* (33%), unclassified *Bradyrhizobiaceae* (11.3%), *Sediminibacterium* (5.8%), *Staphylococcus* (4.3%), *Pseudomonas* (2.3%) FURTD: *Moraxella* (38.6%), unclassified *Bradyrhizobiaceae* (8.8%), *Staphylococcus* (6.3%), *Chlamydia* (5.7%), *Pasteurella* (5.7%), *Sediminibacterium* (4.6%), *Bibersteinia* (4%) Neoplasia: *Moraxella* (15.8%), unclassified *Bradyrhizobiaceae* (20.6%), unclassified *Chitinophagaceae* (7.3%), *Phyllobacterium* (6.6%), *Pasteurella* (5.1%)	No differences between health and diseased cats	(Dorn et al., 2017)

BALF, Bronchoalveolar lavage fluid; BMS, Bronchus mucosal scraping; DNS, deep nasal swap; NPS, nasopharyngeal swaps; OPS, Oropharyngeal swabs; PS, Paranasal sinus; SBT, Segmental bronchi throat; TA, tracheal aspiration; TS, tonsil swabs; WMS, whole metagenome sequencing.

A noticeable and consistent overlap between URT and LRT profile of microbial communities was observed in mammals (**Table 1**). In ruminants, it is likely the result of regurgitating of feedstuffs during rumination and the formation of aerosols during eructation (Klima et al., 2019). Thus, the oral microbiota in cattle probably acts as secondary reservoirs for the lung (McMullen et al., 2020). No information is available on the relationship between the URT and LRT in pigs, and the existing results of LRT microbiota are divergent at the phyla level (Huang et al., 2019; Jiang et al., 2019; Li et al., 2021). In line with the observation in ruminants, studies in cats and dogs support micro-aspiration of pharyngeal secretions as a primary route of microbial colonization of the LRT (Vientós-Plotts et al., 2017; Vientós-Plotts et al., 2019; Fastrès et al., 2020b). In healthy horses, the similarity between nose and lung microbiomes is expected for biological reasons (Bond et al., 2017), such as the large minute ventilation (60 L/min at rest) of these obligate nasal breathers and the fact that they have complete separation of the nasopharynx and oropharynx owing to their long velum, except when swallowing (Fillion-Bertrand et al., 2019). The end result of this interplay is the microbiome of lungs closely resembles that of the oropharynx in the vast majority of healthy mammals. However, it is not clear to what extent the deep airways are stably colonized by specific taxa or whether the microorganisms are in a dynamic state of flux being permanently cleared and repopulated from the URT.

In poultry, Firmicutes, Proteobacteria, Bacteroides, Actinobacteria and Tenericutes are the main well-documented phyla in the upper respiratory (Sohail et al., 2015; Glendinning et al., 2017b; Ngunjiri et al., 2019; Abundo et al., 2021; Kursa et al., 2021) (**Table 1**). In a stable state, the URT is colonized by Bacilli and Clostridia, whereas the LRT, including air sacs, is populated by Bacilli and γ-Proteobacteria (Abundo et al., 2021). However, birds seem to have lost an association with Bacteroidetes but retained an association with facultative anaerobes species from the Proteobacteria phylum (Taylor et al., 2020). The loss of Bacteroidetes likely increases the proportion of transient environmental microorganisms and decreases the observed degree of host specificity and resilience (Taylor et al., 2020). Indeed, this could also help explain the dominance of Proteobacteria, which make up a large proportion of the airborne microbiome in birds (Cáliz et al., 2018), a source to which flighted animals are constantly exposed. At finer grains, birds present fewer obligate anaerobes and more facultative anaerobes compared to mammals. *Lactobacillus* spp. is the main dominant and stable colonizer of the upper respiratory tract (nasal cavity and choanal cleft), albeit varying relative abundances (Sohail et al., 2015; Glendinning et al., 2017b; Johnson et al., 2018; Abundo et al., 2020; Mulholland et al., 2021). *Lactobacilli* are followed by members of the family *Enterobacteriaceae* and of *Staphylococcus* spp. and *Escherichia-Shigella* (Glendinning et al., 2017b; Ngunjiri et al., 2019; Kursa et al., 2021). In the LRT, *Lactobacillus* are also the dominant bacterial taxa (Johnson et al., 2018; Abundo et al., 2021), outranking *Vibrio* and *Halomonas*, as determined by lower respiratory lavage in turkeys, chicken broilers and chicken layers raised in commercial settings (Abundo et al., 2021).

Collectively, these observations support the emerging idea that the nasal cavity in birds, which has the highest microbiota richness, serves as the mainland from which other respiratory sites are colonized (Abundo et al., 2020). The constant movement of fluid and air in the choana can play a major role in equilibrating the microbiome of URT with that of the gut or environment in birds and secondly, it can be responsible for the vast majority of the constant microbial seeding of the lower airways from both oral and nasal cavity. Bacterial overlap between the respiratory tract and gut has been observed in healthy layers raised in commercial farms (Ngunjiri et al., 2019) and turkeys (Taylor et al., 2020; Kursa et al., 2021), possibly through aerosolization of fecal bacteria and the contact with the litter environment.

Other host-related factors that might affect respiratory microbiota variations among mammals and birds involve mucus secretion, gas concentrations, osmolality, temperature, pH, nutrients such as iron and vitamins, surfactant secretion, blood flow, as well as extracellular DNA (Zhu et al., 2019). Additionally, the IgA secretion and the innate and adaptative immune recognition, including the antimicrobial peptides and the sensing of microbes, are endogenous forces that contribute to regulating the host-microbiota interactions in the respiratory system (Wypych et al., 2019).

## Environmental Stressors as Drivers of the Respiratory Microbiota Composition and Dynamics Across Species

**Figure 2:**
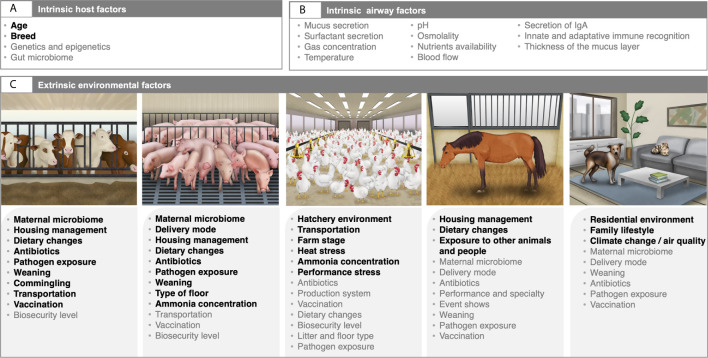
Intrinsic and extrinsic factors affecting the composition and development of the airway microbiota in domestic animals. The respiratory tract microbiota composition and development are dynamic and are shaped by various intrinsic host factors **(A)**, as well as intrinsic airway milieu conditions **(B)**, and environmental variables **(C)**. Factors highlighted in bold are based on published literature. For ruminants and swine, factors are mainly extracted from the review by Zeineldin et al. (2019) and Pirolo et al. (2021), respectively. Variables colored in grey are those that illustrate directional hypothesis made by the authors. Written permission for publication of the domestic animals’ drawings in the figure has been taken, except for the horse drawing. Horse drawing has been obtained from Mach et al. (2020).

In mammalian hosts, the newborn animals are considered sterile *in utero* during a normal pregnancy, but during the first hours of life, a wide range of microorganisms are acquired after the passage through the birth canal, during suckling and maternal care, and from the immediate environment at delivery, such as pen material, feed and feces. Consequently, the microbiota of the calf URT is highly similar to the maternal vaginal microbiota (Lima et al., 2019). Immediately after birth, the airway microbiota in ruminants evolves and reaches a maximum of diversity within one month of age (Timsit et al., 2017; Lima et al., 2019). The taxa organisms detected in the LRT shift from Proteobacteria and Firmicutes to Bacteroidetes and Tenericutes across age (Lima et al., 2019). Such microbiota changes are thought to be intertwined with immune system maturation that promotes tolerance to environmental allergens (Ramírez-Labrada et al., 2020). If the colonization process is disrupted, the animal may develop a dysbiotic microbiota, causing a predisposition to contracting complex respiratory disease. An elegant review by Zeineldin et al. (2019) about ruminants declared that changes in diet (Hall et al., 2017; Maynou et al., 2019), antimicrobial use (Collie et al., 2015; Timsit et al., 2017; Holman et al., 2018; Holman et al., 2019; McMullen et al., 2019), pathogen exposure (Holman et al., 2015), as well as early life management procedures, including weaning, vaccination, commingling, long-distance transportation and housing (Timsit et al., 2016; Holman et al., 2017; Timsit et al., 2017; Zeineldin et al., 2017a; McMullen et al., 2018; Amat et al., 2019) are major contributing factors to the initial seeding disruption of the airway microbiota and have been associated with various health outcomes later in life (**Figure 2**).

Along the same lines, the nasal microbiota of piglets following delivery resembles that of the sow and depends on the route by which the pig is delivered and the feeding type (Wang et al., 2013a). The tonsillar microbiota of piglets following birth resembles the sow vaginal and teat skin microbiota, indicating that these two body sites contribute to the colonization of the URT (Pena Cortes et al., 2018). However, no studies have examined the initial seeding and development of the LRT microbiome in swine. Thereafter, the microbiota begins to stabilize and progress toward and adult-like composition 2–3 weeks after the weaning (Slifierz et al., 2015; Cortes et al., 2018). As previously described in the gut (Mach et al., 2015), weaning is the critical period for the establishment of a robust and stable adult-like microbiota composition in piglets (Pirolo et al., 2021). Alongside weaning, other key environmental factors can modify the composition of the URT microbiota early in life, including the addition or removal of feed antibiotics (Zeineldin et al., 2018; Correa-Fiz et al., 2019; Mou et al., 2019; Zeineldin et al., 2019), gaseous ammonia concentration (Michiels et al., 2015; Wang et al., 2019), type of floor (Megahed et al., 2019) or feeding strategies (Weese et al., 2014).

The way in which different bacteria populate the respiratory microbiota is unknown in horses. Yet, it is not demonstrated that mode of birth, type of diet and antibiotic use have profound effects on the respiratory microbiome of newborn up to some months or years of age in horses. The age at which the respiratory microbiota acquires an adult-like configuration is still unclear. Maternal separation at weaning has a pivotal influence on the gut microbiome composition in foals (Mach et al., 2017). This likely holds true for the respiratory microbiota. Our previous findings in the gut (Mach et al., 2020; Mach et al., 2021a; Mach et al., 2021b) also highlight the possibility that the airway microbiota may be particularly sensitive to where a horse lives and what a horse does. In fact, short-term changes in housing and forage type alters the pulmonary microbiota in horses (Fillion-Bertrand et al., 2019), as well as the contact with animals and people who work at horse facilities, veterinary health care and medication (Kauter et al., 2019).

In a similar manner, published literature on the effects of birth mode on the respiratory microbial composition during the first years of life in companion animals is scant. Whether C-section is associated with lower bacterial diversity and linked with allergy is also unclear. Nonetheless, the combined effect of passive (living environment) and active (lifestyle) factors on the airway microbiota in companion animals from developed countries have started to receive attention for its role in complex respiratory diseases, especially asthma (**Figure 2**). At least, dogs living in urban environments (worse air quality/air pollution) and exposed to an urban-type lifestyle (e.g., living closed in apartments for hours and in a single-person family without other pets) may present an altered microbiota and be more susceptible to respiratory diseases compared to those living in a rural environment and farming lifestyle (Lehtimäki et al., 2018). In agreement with the posited hypothesis, climate change that affects air quality has been shown to lead to alterations in microbial communities in the dog airways (Ericsson et al., 2020). Therefore, the living conditions are suspected to play a role in the airway microbiota in dogs, while no differences are found between types of breeds (Fastrès et al., 2020b). This assumed interplay between urban-type lifestyle, microbial exposure, host microbiota and respiratory disease needs to be confirmed in cats. The information of whether the microbiota of urban cats is more alike than rural ones is still lacking.

Concerning the airway microbiota in birds, the initial colonization of commercially hatched chickens is mainly dependent on the hatchery environment and transport to the farm (Brugman et al., 2018; Kubasova et al., 2019). Notably, some microorganisms can be acquired in the pre-hatching phase, directly from the mother in the oviduct of the hen, or from the environment through the pores in the eggshell (Kers et al., 2018). After birth, lower airway microbiota of commercial chickens kept indoors gradually assembles, without clear separation between brooding, growing and laying stages, while nasal microbiota shifts drastically after the birds transitioned to the laying stage (17 weeks onward) (Ngunjiri et al., 2019). Yet, despite the results by Ngunjiri et al. (2019), little is known about what drives the acquisition and development of the respiratory microbiota during early in life in poultry. Information is scant when focusing on chicks that have any form of contact with adult hen microbiota, chicks that live outdoors throughout their whole life or free-range organic chicken. Exposure to chronic heat-stress (Sohail et al., 2015), atmosphere ammonia concentrations (Liu et al., 2020; Zhou et al., 2021), housing and environmental conditions (Kursa et al., 2021), as well as performance stress (Kursa et al., 2021) are known to shape the composition of the respiratory tract microbiota in domestic birds. As for gut, environmental factors such as biosecurity level, disease onset, litter and feed access may each influence the poultry airway microbiota establishment after hatching (Kers et al., 2018) (**Figure 2**). Antibiotic treatments and vaccinations, which are often administrated *via* spray or eye/nose drops, may also affect the development of the microbiota.

## Frenemies: *Lactobacillus* and *Proteobacteria* in Complex Respiratory Diseases

Host mechanisms for pathobiome regulation in the respiratory apparatus might be different between mammalian animal hosts and birds, but they have in common the aim of both preventing the invasion by pathogens and managing symbiotic microorganisms to maximize the benefits for the host. Precisely, the commensal microbiota may directly inhibit the persistence, transmission and evolution of pathogens through mechanisms such as competing for nutrients, bactericidal activity, metabolic inhibition, biofilm formation, quorum sensing, disruption of signaling molecules, and spatial occlusion (Man et al., 2017; Zhu et al., 2019). Additionally, host systemic and respiratory immune response (Man et al., 2017; Zaneveld et al., 2017; Amat et al., 2019; Budden et al., 2019; Li et al., 2019) may indirectly affect the interaction between commensals and pathogens. None of these mechanisms are wholly effective, but they all reflect common actions by commensal microbiota to neutralize threats to their hosts and, by extension, to themselves.

At this time, several examples of possibly actionable mechanisms of competition and cooperation have been reported in ruminants. For instance, microbiota of the alveolar epithelium in adult sheep synthesizes antimicrobial anionic peptides that quickly bind to and inactivate *Mannheimia haemolytica* (Heidari et al., 2002). In cattle, Amat et al. (2019) suggested that the presence of bacteria from the *Lactobacillaceae* family have a competitive exclusion effect on the BRDC-associated *Pasteurellaceae* family. These results were supported by inhibition assays, in which strains within the families of *Lactobacillaceae, Streptococcaceae*, and *Enterococcaceae* displayed *in vitro* antimicrobial activity (Amat et al., 2019). Additionally, *Lactobacillus* were shown to inhibit the growth of *Mannheimia haemolytica in vitro* (Amat et al., 2017). Taken together, commensal species from the *Lactobacillaceae* family may offer resistance to pathogens directly by producing antimicrobials and beneficial metabolites (e.g., lactic acid and SCFA) or indirectly through keeping cells of the innate and adaptive immune system on the tip of their toes, ready to respond rapidly to pathogenic threats when required.

In pigs, the supplementation with the Lactobacillales *Enterococcus faecium* as probiotic carried direct adsorptive trapping of swine influenza viruses (H1N1 and H3N2 subtypes) by direct physical interaction and by reinforcing innate antiviral defense through mediators such as IL-6, TNF-α, IL-10, IFN-α and TLR-3 (Wang et al., 2013b). Evidence that probiotics may be effective in reducing severity, duration, and incidence of respiratory illnesses in herd-based species was stated as being low to very low (Suez et al., 2019).

While an increased prevalence of *Lactobacillus* is a potential diagnostic signature of eubiosis in ruminants and swine, respiratory disorders in those farm animals are often accompanied by an abnormal expansion of Proteobacteria (**Table 1**). More specifically, γ-Proteobacteria, a phylum containing a wide variety of pathobionts, is frequently enriched in the respiratory tract of infected individuals of cattle and pigs, thereby increasing the likelihood of pathogenic responses. In fact, numerous studies in cattle have linked a remarkable loss in α-diversity to a gain in Proteobacteria during disease. For example, the URT of BRDC-affected calves presented higher abundance of *Moraxella, Streptococcus, Haemophilus*, *Neisseria* (Holman et al., 2015), *Pseudomonas* (Gaeta et al., 2017), *Solibacillus* and *Pasteurella* spp. (Zeineldin et al., 2017a). Within the γ-Proteobacteria, *Pasteurella* spp. was also increased by 1.5 in calves with pneumonia, as diagnosed by ultrasonography, compared with calves without pneumonia (Raabis et al., 2021). The lower α-diversity of the nasopharyngeal and tracheal bacterial communities of cattle with bronchopneumonia likely explained why they were colonized by pathogens such as *Mycoplasma bovis, Mannheimia haemolytica* and *Pasteurella multocida* (Timsit et al., 2018). Parallel work by Lima et al. (2016) demonstrated a marked increase in abundance of the *Mannheimia, Moraxella*, and *Mycoplasma* genera in the URT of three days-old calves coupled to the development of pneumonia and otitis media in calves during the first 60 days of life.

Comparably, a significant gain in the Proteobacteria and loss in Firmicutes, species richness and α-diversity was seen in the nasal microbiota of piglets with Glässer’s disease (Correa-Fiz et al., 2016). Interestingly, the *Pasteurellaceae* was one of the main families responsible for the higher abundance in Proteobacteria, with *Haemophilus parasuis*, being clearly increased, as well as the *Mycoplasmataceae* family (Correa-Fiz et al., 2016). The pro-inflammatory bacteria *Moraxella* spp., was significantly increased in the oropharynx of suckling pigs with porcine respiratory disease (Wang et al., 2018), whilst Proteobacteria such as *Sphingobium*, *Haemophilus* and *Phyllobacterium* co-occurred with *Mycoplasma* and *Ureaplasma* in lungs with severe lesions.

Consistent with results observed in pigs and cows, horses infected with equine herpesvirus 1 (EHV-1) had lower nasal bacterial richness, evenness and diversity than healthy horses, conjointly with higher abundance of Proteobacteria (Gomez et al., 2021).

Broadly, a sustained increase in abundance of the phylum Proteobacteria seems a biomarker of dysbiosis in the respiratory tract of cattle, pigs and horses. Whether Proteobacteria are flourishing better when the immune system is stimulated, or whether they actively contribute to the host immune pathology under pathogen infection has still to be explored. As Proteobacteria can tolerate oxidative stress (Million and Raoult, 2018), their expansion is likely linked to the onset of mucosa inflammation following primary infection. Additionally, they encode the metabolic capacity to utilize inflammatory byproducts to survive and prosper under acid and low oxygen conditions (Huffnagle et al., 2017), outcompeting bacteria that lack the metabolic capacity to benefit from inflammation. However, the phylum Proteobacteria, named after the Greek god Proteus, has the largest phylogenetic structure, including 116 validated families of bacteria (Shin et al., 2015). As the name suggests, this phylum presents a large range of morphologies and versatile functions (Shin et al., 2015). Indeed, in cats and dogs, *Pseudomonas* and *Acinetobacter*, microorganisms belonging to the phylum Proteobacteria, probably exert a protective function in the lung of companionship animals **Table 1**). Bacteria in those two genera likely have multiple high-affinity adhesins that mediate binding and biofilm formation preventing the growth of potential pathogens in the lung of cats and dogs (Vientoos-Plotts et al., 2017; Fastrès et al., 2020b). In addition, *Acinetobacter* could play an anti-inflammatory role in the lungs (Fastrès et al., 2020b). Despite arguments regarding beneficial functions of Proteobacteria, a blooming pattern of Proteobacteria following disruption of homeostasis by environmental or host factors can further facilitate inflammation or invasion by exogenous pathogens in mammals.

Yet, the nature of Proteobacteria in the respiratory pathobiome in poultry remains largely unknown. Only Liu et al. (2020), have recently evoked a relationship between the increase of Proteobacteria under seven days of ammonia exposure and respiratory tract diseases in broilers.

## Complex Respiratory Disease and Comorbidities: The Multiple Pathogen Disease Paradigm in a Pathobiome Context

As central finding from respiratory tract microbiota is that most complex airway diseases are caused by pathogens that can be common members of a symbiome in the absence of disease. The well-characterized BRDC is an example of how commensal inhabitants of the respiratory tract (namely *Mannheimia haemolytica, Histophilus somni, Pasteurella multocida, Trueperella pyogenes, Mycoplasma bovis, Arcanobacterium pyogenes, Mycoplasma dispar, Ureaplasma diversum*, and *Mycoplasma bovirhinis)* proliferate in the URT and invade the lung *via* inhalation under specific conditions (Griffin et al., 2010). Likewise, the URT of clinically asymptomatic pigs often harbors pathobionts such *Mycoplasma hyopneumoniae, Haemophilus parasuis, Actinobacillus pleuropneumoniae, Actinobacillus suis, Pasteurella multocida, Bordetella bronchiseptica* and *Streptococcus suis* (Luehrs et al., 2017; Vötsch et al., 2018) that can all prone to disease. In dogs, many potential pathogens involved with CIRDC*, e.g.*, *Mycoplasma cynos*, *Bordetella bronchiseptica* and *Streptococcus* spp. are common members of the respiratory microbiota (Day et al., 2020). Similarly, the URT is also a reservoir of opportunistic pathogens in domestic birds. Potential respiratory pathogens, namely *Avibacterium, Gallibacterium, Mycoplasma*, and *Ornithobacterium*, are found in the URT of turkeys (Kursa et al., 2021).

The next complexity that we can bring in is that the modifications of the airway microbiota resulting from primary virus infections often act as a disturbance for secondary infections (Zeineldin et al., 2019). The one pathogen-one disease paradigm is often insufficient to elucidate many of the respiratory diseases (Vayssier-Taussat et al., 2014; Vayssier-Taussat et al., 2015).

A plethora of examples in swine illustrates the framework in which co-infection occurs. Swine influenza virus enhances the morbidity of *Streptococcus suis* infection by decreasing mucociliary clearance, damaging epithelial cells, and by facilitating its adherence, colonization and invasion in the lungs (Meng et al., 2016). Swine influenza virus also compensates for the lack of suilysin (cytotoxic protein secreted by *Streptococcus suis*) in the adherence and invasion process of suilysin-negative *Streptococcus suis* (Meng et al., 2019). Similarly, a synergism between nasal *Staphylococcus aureus* and pathobionts such as *Pasteurella multocida* and *Klebsiella* spp. have also been reported in pigs (Espinosa-Gongora et al., 2016). Co-occurrence between the porcine reproductive and respiratory syndrome virus (PRRSV), *Haemophilus parasuis* and *Mycoplasma hyorhinis* in pig lungs is repeatedly observed (Jiang et al., 2019). Contrastingly, the virulence of one pathogen may be reduced by co-infection with another, as it has been shown for the bacterium *Escherichia/Shigella* in the respiratory tract of weaning piglets, which inhibited the virulence of *Haemophilus parasuis* or the presence of *Phascolarctobacterium* in the respiratory tract (Mahmmod et al., 2020). On the host side, respiratory pathogens can also modulate innate and adaptive immune responses (e.g. weakening monocyte activity, suppressing phagocytic capacity of alveolar macrophages, among others) that therefore promote secondary bacterial infection (Zeineldin et al., 2019). For example, PRRSV infection of bone marrow-derived dendric cells regulated the innate immune system by partially acting on the phagocytosis of *S. suis*, but mainly by modulating the development of an exacerbated inflammatory response (Auray et al., 2016). Similarly, *in vitro* co-infection of swine epithelial cells with *S*. *suis* and swine influenza virus showed an important synergy between the two pathogens regarding the up-regulation of genes coding for inflammatory mediators (Dang et al., 2014). Cells co-infected with PRRSV and porcine circovirus produced more TNF than cells infected with just one of the pathogens (Shi et al., 2010), whereas an exacerbated inflammatory response was described in pigs co-infected with PRRSV and *Mycoplasma hyopneumoniae* (Thanawongnuwech et al., 2004).

Another case in point are the low pathogenic avian influenza viruses (LPAIV) that, during outbreaks, even in the antibiotic era, are coupled to co-infections by pathogens such as *Mycoplasma gallisepticum, Mycoplasma synoviae*, *Ornithobacterium rhinotracheale*, avian pathogenic *Escherichia coli* (APEC) and *Staphylococcus aureus*, which are responsible for a higher mortality rate (Much et al., 2002; Belkasmi et al., 2020) and a marked reduction in egg production of laying hens (Umar et al., 2016). Moreover, it appears as though that concurrent pathogens such as infectious bronchitis virus (IBV), the APEC, the avian pneumovirus and the LPAIV act synergistically or cumulatively to mediate complex infectious processes (Awad et al., 2014; Guabiraba and Schouler, 2015; Patel et al., 2018).

## The Gut-Lung Axis During Respiratory Diseases: Key to Understanding Holobiont Functioning

The pathophysiology of the complex respiratory diseases in domestic animals seems more complex than previously assumed. It has been recently posited that respiratory comorbidities might be partly modulated by the bidirectional inter-organ communication with the gastrointestinal tract, referred to as the gut-lung axis (Dang and Marsland, 2019). This new field of research is now investigating how gut microbiota modulates the onset of respiratory infections (Niederwerder, 2017) and whether the airway microbiota in turn, influence host epithelial and immune cells to adjust inflammatory responses at distal sites such gut (Budden et al., 2017; Wypych et al., 2019).

**Figure 3:**
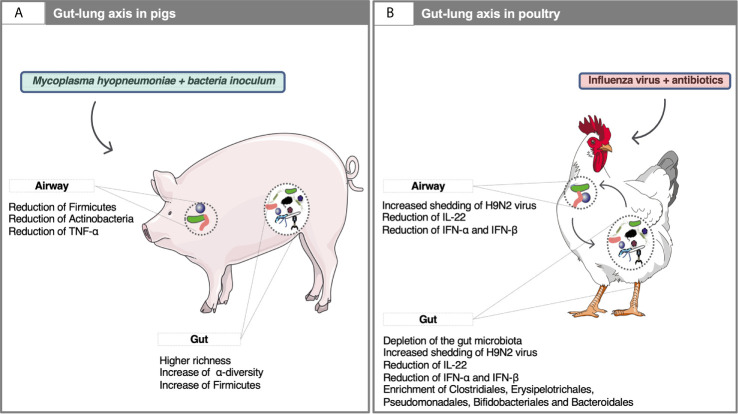
Examples of the gut-lung axis cross-talk in domestic animals. **(A)** The gut microbiota composition affects systemic immune responses and lowers the severity of *M. hyopneumoniae* infection in the respiratory tract of pigs. For example, the administration of oral microbial inoculum increased Firmicutes such as *Roseburia, Barnesiella, Blautia, Dorea* in the gut and reduced the relative abundance of Actinobacteria and Firmicutes phyla in the airways microbiota, as well as the levels of TNF-α in the lungs and their lesions (Schachtschneider et al., 2013). Young pigs with low microbial diversity in the gut showed severe lung lesions on exposure to *M. hyopneumoniae*, whereas the opposite trend was observed in piglets with higher SCFA producing taxa in the gut (*e.g.*, *Ruminococcus*, *Prevotella*, *Ruminiclostridium*, and *Oscillospira*) (Surendran Nair et al., 2019). In addition to having local effects in the gut, SCFAs enter the circulation, modulate bone-marrow hematopoiesis and thereby can promote regulatory or pro-inflammatory responses in the lung (Surendran Nair et al., 2019); **(B)** In H9N2 subtype LPAIV infected chickens, microbiota gut depletion using antibiotics have shown to reduce IFN response, which plays an important role in innate responses to viral infections in the gut and airways and increase the influenza virus shedding from the upper respiratory and gastrointestinal tracts (Yitbarek et al., 2018a). Along with IFNs, treatment of chickens with antibiotics for 12 days resulted in reduced interleukin 22 expression in the respiratory tract after gut microbiota depletion and in enrichment with class Erysipelotrichia, Bacteroidia and with order Clostridiales, Erysipelotrichales, Pseudomonadales, Bifidobacteriales and Bacteroidales (Yitbarek et al., 2018b). Written permission for publication of the chicken drawing in the figure has been taken. The pictures of the pig, bacteria and viruses were downloaded from smart Servier Medical Art https://smart.servier.com without changes. Servier Medical Art by Servier is licensed under a Creative Commons Attribution 3.0 License.

The gut of ruminants (Ramayo-Caldas et al., 2019), pigs (Mach et al., 2015; Ramayo-Caldas et al., 2016), horses (Mach et al., 2020; Mach et al., 2021a), companion animals (Alessandri et al., 2020) and chickens (Rychlik, 2020) accommodates a complex community of microorganisms that live in a commensal relationship with their hosts and provide significant contributions to the metabolism and immune responses. It might be hypothesized that in symbiosis, gut microbiota ensures the uptake of microbial metabolites that favorably affects the immune and endocrine pathways involved in respiratory disease and progression, whereas the lungs maintain inflammatory homeostasis in the gut by controlling immune response. Even if the mechanisms bridging gut microbiota with the alterations of respiratory disease outcome are still poorly understood in domestic animals, a growing body of research in swine supports that the crosstalk between the gut and airways microbiome. The gut microbiome composition in growing pigs following an exposure to a non-pathogenic oral microbial inoculum modulated microbial communities and was beneficial upon challenge with *Mycoplasma hyopneumoniae* (Schachtschneider et al., 2013). Similarly, the gut microbiome diversity and composition in piglets determined the respiratory disease progression in pigs after *M. hyopneumoniae* inoculation [Surendran Nair et al. (2019); **Figure 3A**]. Likewise, increased fecal microbiome diversity was associated with improved health outcomes after experimental co-infection with PRRSV and porcine circovirus type 2 (PCV2) (Niederwerder et al., 2016; Ober et al., 2017; Niederwerder et al., 2018).

Several recent studies in domestic birds have also tackled this broader question of the gut-lung axis. Antibiotic treatment in day-old layer chickens resulted in a significant depletion of the gut microbiota, a down-regulation of the type I interferon response and higher shedding of LPAIV of H9N2 subtype in the oropharynx and cloacal section (Yitbarek et al., 2018a). A similar study (Yitbarek et al., 2018b) reported that depletion of the gut microbiota in young chickens using antibiotic treatment increased both oropharyngeal and cloacal levels of the LPAIV of H9N2 subtype and decreased the expression of IFN-α and IFN-β in the gut and respiratory tract (**Figure 3B**). Likewise, depletion of gut microbiota increased the severity of Marek’s disease in infected chickens, coupled to an increase in the transcription of IFN-α, IFN-β and IFN-γ in the bursa of *Fabricius* at four days post-infection (Bavananthasivam et al., 2021). In another study developed in chickens, *Lactobacillus salivarius* intake alleviated lung inflammation injury caused by *Mycoplasma gallisepticum* infection and increased host defense against *Escherichia coli* by improved gut microbiota composition (Wang et al., 2021). Antibiotic-treated ducks had increased levels of intestinal highly pathogenic avian influenza virus (HPAIV) of H5N9 subtype associated with a reduced antiviral immune response, but no higher viral titers in the respiratory tract (Figueroa et al., 2020). Finally, the oral administration of *E. coli* O86:B7 expressing high levels of galactose-α-1,3-galactose (α-Gal) in turkeys was found to abrogate anti-α-Gal IgA response in lungs and to protect against experimental *Aspergillus fumigatus* infection (Mateos Hernández et al., 2020). The authors suggested that gut microbiota generates α-Gal-specific antigen-specific regulatory T cells (Tregs) in the gut, which can then migrate to the lungs, promoting disease tolerance which prevents the systemic upregulation of pro-inflammatory cytokines (Mateos Hernández et al., 2020).

As outlined above, knowledge regarding the crosstalk mechanisms by which the airway microbiota affects immune response in the gut is scant (Brown et al., 2017; Mazel-Sanchez et al., 2019). It is postulated that gut microbiota can reach occasionally the lungs through aspiration of vomit or esophageal reflux, therefore exposing pattern-recognition receptors (PRRs) expressed by host cells to peptidoglycans or lipopolysaccharides and stimulating the immune response. Alternatively, these microbe-associated molecular patterns can be transported *via* the circulation together with other gut-derived microbial metabolites known to have immunomodulatory properties such as short-chain fatty acids (SCFA) or indole derivates (Maslowski et al., 2009; Wypych et al., 2019). Avian influenza virus infections have shown to alter the intestinal microbiota in domestic poultry and aquatic birds (Zhao et al., 2018). However, these alterations are likely the outcome of the viral replication in the avian intestinal tract, rather than a cross-talk between respiratory and intestinal mucosal surfaces.

## The Zoonotic Potential of Influenza A Viruses: A Crawling Threat

Because species of veterinary interest are in regular and increasingly close contact with humans, especially in developing countries, understanding the environmental and host determinants that allow pathogens to develop in the respiratory system of livestock and occasionally to be transmitted to and cause diseases in humans is also a relevant research questions today (Peiris et al., 2012). Influenza A viruses, which are commonly found during the onset of complex respiratory diseases in livestock, highlight the problem of devastating zoonotic infections that can arise from veterinary species such as avian and mammalian animal hosts (Kuiken et al., 2005; Mostafa et al., 2018). For instance, in one study involving 125 farrow-to-finish pig hers in France, Influenza A viruses of H1N1 subtype was reportedly found during respiratory disorders (Fablet et al., 2012). Of particular concern is the emergence of the LPAIV of H7Nx subtypes infecting humans, which are frequently reported in commercial poultry, backyards and live bird markets in numerous countries in Europe, Asia and Africa (Abdelwhab et al., 2014; Richard et al., 2014). While H7N2, H7N3 and H7N7 subtypes LPAIV occasionally infect humans causing mild, if any, clinical manifestations (Gao et al., 2013), the emerged H7N9 subtype LPAIV in China causes a high mortality rate in humans (40%) and have pandemic potential (Horman et al., 2018). Additionally, a growing threat to both animal and human health is emerging from the H9N2 subtype LPAIV, endemic in poultry in many areas of Eurasia and Africa (Peacock et al., 2019).

## Conclusions

The last decade has brought substantial insights into the characterization of the respiratory microbiota in species of veterinary interest. Sterility of the airways has been rejected. The mechanisms behind the colonization of the respiratory system in the different species have started to be explored, as well as the microbial communities occupying the different niches in the airways. Yet nothing is known about the role eukaryotes, archaea and phages play in the respiratory tract in domestic animals.

Collectively, the results obtained in the last years suggest some degree of convergence in the airway respiratory microbiota of mammals. At the phylum level, mammals harbor Firmicutes, Bacteroidetes and Proteobacteria in the upper and lower respiratory system. This is juxtaposed with domestic birds, in which we observed a reduction of Bacteroidetes but increased abundance of Proteobacteria, sustaining the host-specific microbe associations. Although these differences are not yet fully understood, the hypothesis that birds are merely “feathered” mammals with a few specific differences related to flight and production of large yolky eggs is an erroneous assumption. Therefore, not all of the results obtained on one species can be directly translated to the other ones.

The increase in the prevalence of respiratory disorders in farm and companion animals have reached epidemic statistical levels in wealthy countries that generate a greater need to monitor and control their impact on morbidity and mortality. The increasing demands for livestock products coupled to the intensification of marked-oriented systems in many countries, climate change, ecology incursion, as well as movements of people, animals and products increase the risk of complex respiratory disease development worldwide (Kuiken et al., 2005), even if data for regions in South America, Asia, Middle-Est and Africa is scant. The prevention and treatment of respiratory diseases are of paramount importance, not only because they can induce immunosuppression and render animals susceptible to opportunistic infections, but also because it may increase the risk of cross-species transmissions, foodborne and zoonotic diseases.

Disorders in the respiratory tract are compatible with a “ménage à quatre” situation, as they depend on the intricate interactions between the pathogen(s), multiple symbionts of the respiratory tract, the host response and the environmental conditions. There is evidence showing that various environmental factors such as abrupt weaning, antibiotic administration and housing management often disrupt the respiratory microbial ecosystem and increase susceptibility to infections in animal species used for food. Under unfavorable environmental conditions, the symbionts themselves can act as opportunistic pathogens or not provide the same degree of protection. An example hereof is that the respiratory disease complex in bovine, porcine and commercial birds often imply pathobionts. Pigs, ruminants and birds, which frequently switch environments through their intense production lives, are more prone to exhibit respiratory disorders. Modulation of husbandry environment may facilitate the manipulation of the pathobiotic systems. Beyond farm animals, urbanized lifestyle, featuring restricted animal contact and house confinement promotes changes in airway microbiota in both companion animals and horses.

The commensal microbiota composition may affect the pathogen infectivity or the expansion of pathobionts beyond a level of tolerance, either directly *via* the secretion of molecules with antimicrobial activity or indirectly *via* immune-mediated modifications. Of the remaining questions are: which taxa or mixtures of these, if any, make animals more susceptible or resistant to respiratory disorders in each of the domestic species. Thus far, data in mammals have identified members of the *Lactobacillaceae* family as key protective hub taxa in the respiratory tract. Conversely, the expansion in Proteobacteria coupled with a drop in Firmicutes and in diversity has been associated with a pathobiome context in cows, pigs and horses. Proteobacteria seem benign when they are in minor proportion, whereas, under the onset of disease, they trigger inflammatory responses.

The blooming of Proteobacteria could be a potential biomarker for respiratory disease states in mammals and birds, however, trends and nuances are noted. Species within the Proteobacteria phylum have a protective role in immune response against infection or inflammation, as shown in companionship animals. Therefore, one can speculate that bacteria from the phylum Proteobacteria have as yet unidentified functions, and so a better understanding of such functions is key to identifying their symbiotic or their pathological relations with hosts.

To date, re-establishment of respiratory eubiosis after infections and preventive treatment with probiotics has not been demonstrated in domestic animals. Nonetheless, manipulation of gut microbiota in swine and domestic birds has shown to shape their immune response and interfere with the course of respiratory disease. The existence of a gut-lung axis paves the way for new approaches in the management of respiratory disease in domestic animals.

## Future Perspectives

Despite significant efforts towards understanding the link between the pathogen(s), the commensal host-microbiota and the environment, this research field has just started to explore the mechanisms by which pathobiome disrupts respiratory homeostasis in animals. An improved understanding of the spatial distribution and temporal changes from a commensal microbiota to a pathobiome state would provide valuable information (biomarkers) to implement preventive measures or treatments, before observation of clinical signs or symptoms. Both symbiome and pathobiome vary over time and between individuals. Thus, longitudinal studies focusing on the aforementioned quadrangular interactions are needed to understand the role that the symbiome and pathobiome may have in agent-associated disease outbreaks, as well as the forces at play. These should concentrate on gaining a better understanding of the shifts of the microbiota composition and function in different sections of the respiratory and gastrointestinal systems, and whether the microbiota shifts occur before, and cause a change in respiratory health status, or if they are a consequence of a change in health due to a different host and environmental factors. Expanding shotgun metagenomic sequencing and functional metagenomics (*e.g.*, metatranscriptomics, metaproteomics, metabolomics) of the respiratory tract is required to address this important issue. Advances in sequencing and database annotation are critical for further advances in the field that have the potential to optimize the methods to mitigate diseases, including those related to emerging zoonotic pathogens.

## Author Contributions

NM designed the structure of this review and wrote the manuscript. EB, LN, and CC have contributed to the reviewing and editing of the manuscript. All authors contributed to the article and approved the submitted version.

## Funding

This work was supported by financial supports from INRAE.

## Conflict of Interest

The authors declare that the research was conducted in the absence of any commercial or financial relationships that could be construed as a potential conflict of interest.
